# Chlorine Distribution in Soil and Vegetation in Boreal Habitats along a Moisture Gradient from Upland Forest to Lake Margin Wetlands

**DOI:** 10.1021/acs.est.2c09571

**Published:** 2023-07-20

**Authors:** Teresia Svensson, Anders Löfgren, Peter Saetre, Ulrik Kautsky, David Bastviken

**Affiliations:** † Department of Thematic Studies - Environmental Change, Linköping University, 581 83 Linköping, Sweden; ‡ EcoAnalytica, Slalomvägen 28, 129 49 Hägersten, Sweden; § Swedish Nuclear Fuel and Waste Management Co. (SKB), P.O. Box 3091, 169 03 Solna, Sweden

**Keywords:** chloride, retention, discharge area, Cl_org_, chlorination, residence time, vegetation, ecosystem, Cl-36

## Abstract

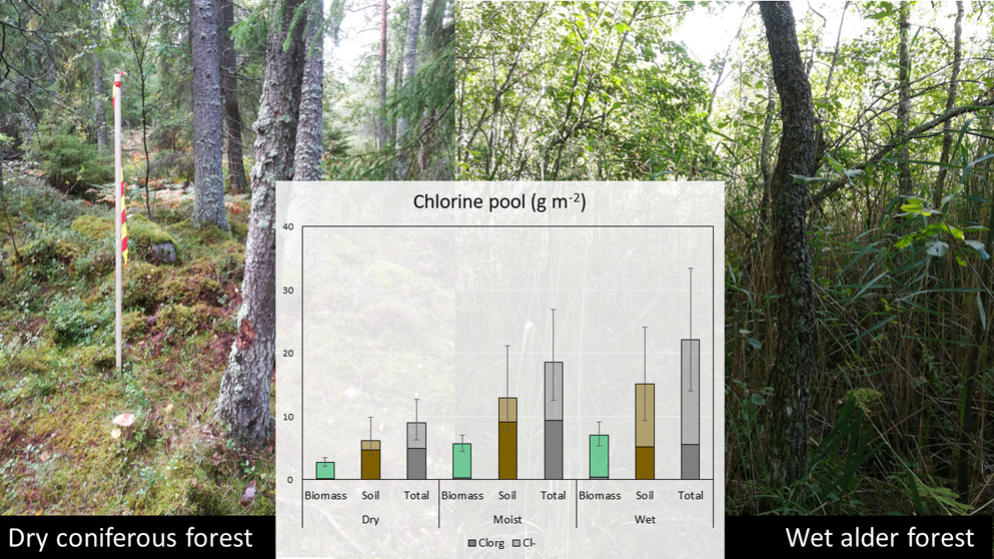

The assumed dominance of chloride (Cl^–^) in terrestrial ecosystems is challenged by observations of extensive formation of organically bound Cl (Cl_org_), resulting in large soil Cl storage and internal cycling. Yet, little is known about the spatial distribution of Cl in ecosystems. We quantified patterns of Cl distribution in different habitats along a boreal hillslope moisture gradient ranging from relatively dry upland coniferous forests to wet discharge areas dominated by alder. We confirmed that dry habitats are important for Cl storage but found that Cl pools tended to be larger in moist and wet habitats. The storage of Cl_org_ was less important in wet habitats, suggesting a shift in the balance between soil chlorination and dechlorination rates. Cl concentrations in the herb layer vegetation were high in wet and moist sites attributed to a shift in plant species composition, indicating plant community-dependent ecosystem Cl cycling. Mass-balance calculations showed that internal Cl cycling increased overall ecosystem Cl residence times at all sites and that plant uptake rates of Cl^–^ were particularly high at wet sites. Our results indicate that habitat characteristics including plant communities and hydrology are key for understanding Cl cycling in the environment.

## Abstract 2

Interactions between plant communities and Cl biogeochemistry along hydrological gradients across landscapes are fundamental knowledge for interpreting most Cl-related environmental studies.

## Introduction

Chloride (Cl^–^), the predominant form of chlorine (Cl) in nature, was previously considered nonreactive in terrestrial ecosystems but this view has changed.^1−3^ Extensive natural chlorination of organic matter has been observed in many terrestrial ecosystems.^4−7^ Experiments with radioactive Cl (^36^Cl) as a tracer have confirmed natural chlorination rates corresponding to as much as 50–300% of the annual wet deposition of Cl in several types of soils without plant roots,^8,9^ and substantially higher organic matter chlorination rates with active roots present.^10^ The variation of soil Cl at large and coarse spatial scales has been correlated to atmospheric Cl^–^ deposition and soil organic matter content.^4,5,11,12^ At local scales, other factors may be more important, but detailed Cl distribution among local topography causing gradients in soil wetness, nutrient flux, and biogeochemical cycling in the landscape have rarely been assessed.

Studies on soil Cl cycling published to date have primarily focused on relatively dry recharge areas, such as upland forests and agricultural land.^13^ Little is known regarding Cl cycling in discharge areas and wetland habitats. Such areas are known key sites for element transit and transformation processes due to the high biological activity combined with abundant redox gradients.^14,15^ Discharge areas are also of particular interest to determine the fate of compounds reaching surface ecosystems through groundwater, which is of major concern for planning future subsurface repositories of nuclear waste where a leakage of ^36^Cl, with a half-life of 300000 years, is likely to reach the biota in discharge areas. Cl can be retained as organically bound Cl (Cl_org_) in surface soils or as Cl^–^ in biomass and there seems to be a continuous cycling between these Cl pools. Montelius et al.^16^ could link Cl^–^ deposition over 30 years to the accumulation of Cl_org_ in forest soils at rates strongly linked to dominant forest tree species. Thus, to understand landscape Cl cycling and residence times, it is necessary to characterize Cl^–^ and Cl_org_ uptake and storage in common habitats and vegetation communities along gradients. This is of fundamental importance for the use of Cl^–^ for assessing subsurface water movement and catchment hydrology,^17,18^ and for risk assessment modeling associated with ^36^Cl in nuclear waste^19,20^ and other pollutants carried by groundwater and soil water.^13−15^

Forest growth usually changes with the hillslope position, with the greatest production found at the low-end position, which is typically characterized by a lush and diverse herb layer.^21^ The increased productivity is likely to be due to an increased availability of water, macronutrients, and base cations in the discharge areas, compared to associated uplands.^22^ Increased forest growth could lead to a larger accumulation of Cl in biomass and a larger input of Cl to topsoil from plant litter.^23,24^ If Cl is taken up from deep soil by vegetation and becomes redistributed via litter to the biologically active surface soil layers, this can potentially affect whole-system Cl cycling. Biotic chlorination is generally assumed to be the primary process for the formation of Cl_org,_^8^ and chlorination of soil organic matter has been associated with a low pH optimum.^25^ Moreover, nitrogen availability can hamper chlorination of organic matter,^26,27^ and chlorination is an oxidative process and therefore it may be less extensive in water-logged environments where anoxic reducing conditions are more common such as in discharge areas. On the other hand, areas of high productivity have probably greater availability of labile carbon, a fraction that decomposes relatively rapidly and serves as a readily available energy source, which is stimulating chlorination of soil organic matter.^27^ Hence, it is possible that environmental conditions in discharge areas will have a significant but yet unknown net effect on the balance between Cl_org_ and Cl^–^. The combination between the relative abundance of Cl_org_ vs Cl^–^ and the uptake of Cl in vegetation is key for understanding Cl cycling in terrestrial habitats.

The aim of this study was to analyze the distribution of Cl^–^ and Cl_org_ in vegetation and soil along four hillslope moisture gradients in eastern Sweden. The sampling sites, ranging from upland coniferous forest to wet alder forest, were also characterized with respect to plant species composition, tree, and understory biomass, forest floor mass, and soil chemical characteristics. Based on previous studies, we hypothesize an increasing importance of plant uptake of Cl^–^ with increased wetness along the hillslope gradient, to reflect higher productivity in moist and wet habitats and a transition from a dominance of Cl_org_ bound to soil particles in upland soils to a dominance of Cl^–^ in soil water and biomass in discharge areas, which in turn could have implications for the mobility and residence times of Cl in different hillslope habitats.

## Materials and Methods

### Study Area

The sampling was conducted in Forsmark nearby the Baltic Sea coast in eastern Sweden (60° 23′ N, 18° 11′ E). The average annual precipitation (2003–2010) is 584 mm, the annual average potential evapotranspiration (PET) is 511 mm, and the average yearly temperature is +6.7 °C.^28^ The landscape is dominated by coniferous forest (primarily Scots pine, *Pinus sylvestris*, and Norway spruce, *Picea abies*), and also includes mires and shallow lakes.^29^ The land cover (water excluded) is characterized by 70–75% forest, 10–20% wetlands, 5% arable land, and 5% pasture and meadow.^29^ The estimated vegetation period in the area is April–September.^29^ The area has been rising gradually from the Baltic Sea during Holocene following continental uplift after the last great glaciation period and the ground therefore has a mixed marine and terrestrial origin influencing groundwater chemistry.

### Site Characterization and Survey Designs

The field study was conducted as a hillslope gradient study (Table S1). Four locations were chosen, and at each location, three habitat sampling sites were established along an elevation gradient. The three sites along each gradient were chosen to represent recharge (dry) and discharge (wet) areas at the low end as well as zones with intermittent recharge and discharge (typically moister soils and therefore denoted as moist). The vegetation of the different habitats ranged from spruce forest of bilberry type, spruce forest of low herb type to wet alder forest of herb type (detailed vegetation characteristics in the Supporting information, Table S2).

### Vegetation and Soil Sampling of Hillslope Gradient

For each vegetation layer (excluding trees), a bulk sample of all biomasses in a square (1 m^2^) was collected for chemical analyses (Table 1). If possible, in terms of enough sample biomass being present, subsamples were also collected separately for the dominating species. Green leaves and stems for dwarf shrubs were not separated. For the ground vegetation layer, the recent biomass of moss from the summer was collected separately from the total bulk sample. The tree representing the dominating tree species closest to the square was chosen for the sampling of foliage and wood. Foliage, annual shoot for spruce, and wood sample were collected in PE bags and stored in a refrigerator until further analysis. The largest tree at the site was used for age determination.

**Table 1:** Summary of Sampling for Chemical Analysis^a^

	type of sample		sampling
vegetation	tree	foliage, current year shoots	<2 m from the plot (sample tree)
		phloem	<2 m from the plot (sample tree)
		wood (tree core)	<2 m from the plot (sample tree)
	shrub	stem and foliage	plot (1 × 1 m)
	herb layer	herbs, grasses, and dwarf shrubs (stem and foliage).	plot (1 × 1 m)
	ground layer	biomass and annual shoot	plot (1 × 1 m)
soil	litter layer		plot (1 × 1 m)
(5 random subsamples per plot)	humus layer		plot (1 × 1 m)
	mineral layer		plot (1 × 1 m)

a Habitat representative sampling square of 1 m^2^ was chosen close to a tree at each sampling site (*N* = 1). See text for details on the sampling scheme.

Soil samples for chemical analyses were collected by a soil corer (2.5 cm diameter, 40 cm) at five randomly selected spots within the square and separated into the litter layer, the humus soil layer, and the mineral soil (≤40 cm) (Table 1).

### Chemical Analyses

The soil pH was measured by adding fresh soil to media consisting of deionized water (18 MOhm cm^–1^), KCl (1 mol L^–1^), and CaCl_2_ (0.01 mol L^–1^) (1:5, soil:solution).^30^ Soil for other analyses was freeze-dried (−54 degrees, 48 h) and thereafter homogenized using a mortar. Measurements of Cl were made using analysis procedures for total chlorine (Cl_tot_) as well as total organic chlorine (Cl_org_) according to Asplund et al^4,31^ using a Total Halogen Analyzer (TOX, ECS3000 analyzer, Euroglas). Sieved and milled soil^4^ (approximately 20 mg of sample) was combusted under a stream of oxygen at 1000 °C (Euroglas AOX Analyzer), converting all chlorine to chloride being trapped in a solution. Thereafter, chloride in this solution was determined by microcoulometric titration. For Cl_org_, in short, 20 mg of sample was added to an acidic nitrate solution (0.2 KNO_3_, 0.02 M HNO_3_) and shaken for one hour on a rotary shaker (180 rpm) to extract leachable chlorine from the soil. The solution was then filtered through a polycarbonate filter and rinsed with a nitrate solution (0.01 KNO_3_, 0.001 M HNO_3_), followed by acidified deionized water (18 MOhm cm^–1^, pH < 2). The filter and the soil sample were then combusted and analyzed following the procedure for TX. The results are expressed as μg Cl g^–1^ dry mass. The Cl_org_ concentration may also include mineral-bound Cl (Cl_mineral_). Thus, to quantify the amount of Cl_mineral_ in soil, a separate analysis was made. In this analysis, soil samples were precombusted (at 500 °C for 4 h) to remove organic matter and then leached with acidic nitrate solution to remove all nonmineral chloride prior to the TX analysis procedure. The Cl_mineral_ was then subtracted from the TOX results to yield Cl_org_ in these samples. Cl^–^ (along with small amounts of leachable Cl_org_) was calculated by subtracting values of Cl_org_ and Cl_mineral_ from the TX results.^16^ The fraction of water-leachable Cl has previously been shown to consist of >90% chloride (>99% in the O horizon), <10% Cl_org._^9,31^ Determination of the elemental content of carbon (C), nitrogen (N), and hydrogen (H)—referred to as CNH analysis—was conducted using an elemental analyzer (PerkinElmer EA2400). 0.002–0.015 g of sample was weighed in tin capsules, combusted at 925 °C, and the CNH content was determined. The vegetation samples were freeze-dried in 48 h and thereafter homogenized until further analyses. The vegetation samples were analyzed for total organic halogens (TOX) and total halogens (TX) using the same procedure as for soil samples. The mineral fraction of chlorine (Cl_mineral_) is insignificant in biomass, and TOX for biomass corresponds to the organic fraction Cl_org_. The chloride concentrations were estimated by subtracting TOX from TX. Determination of CNH was conducted using an elemental analyzer by the same procedure as for the soil samples.

### Chlorine Pool and Transfer Rate Estimates

The Cl pool in trees was calculated by multiplying the measured biomass per m^2^ of foliage and stem (basal area weighted) with the measured Cl concentrations of stem and foliage sample of the sample tree (Table 2). The Cl pool in the understory was calculated separately for the shrub, herb, and ground layers. For each layer, the measured Cl concentrations of the vegetation were multiplied with the measured biomass per m^2^. Soil Cl pools were calculated by multiplying the average Cl concentrations with the mass per m^2^ for each soil layer. The soil mass per square meter was calculated by multiplying the soil bulk density with the average thickness of the soil layer, accounting for the content of stones and boulders.^32^ For each habitat, the transfer rates from the soil Cl^–^ pool to each biomass pool were calculated as described in the Supporting information. See Results and Discussion for proposed rate constants.

**Table 2:** Biomass, C/N Ratio, pH, and Soil Water Content at the Investigated Habitats^a^

	dry	moist	wet	*p-value*
biomass foliage (kg m^–2^)	2.6 a	3.2 a	1.3 b	0.001
biomass wood (kg m^–2^)	22	31	39	0.12
biomass herb layer (kg m^–2^)	0.07	0.07	0.12	0.47
biomass ground layer (kg m^–2^)	0.28	0.17	0.08	0.27
litter mass (kg m^–2^)	3.9	4.8	6.0	0.25
litter C (%)	48	46	47	0.18
litter C/N ratio	36	36	27	0.15
humus mass (kg m^–2^)	9.0	36.1	27.6	0.13
humus C (%)	43	24	30	0.23
humus C/N ratio	27 a	22 b	16 c	0.005
soil water content (%)	57%	58%	73%	0.13
pH (KCl)	3.2 a	4.5 a	6.0 b	0.01

a Average values are shown for each hillslope habitat (*N* = 4), and values with different letters differ in a pairwise comparison with a significance level of *p* < 0.05 (Student’s *t*-test). Dry: upland dry coniferous forest, moist: moist coniferous forest, and wet: wet alder forest wetland. The *p*-value gives the probability that the three habitats have a common mean, corresponding to the test of the factor habitat in the statistical model (see Statistical Analysis for details).

### Statistical Analysis

To test for patterns between habitats and Cl concentration (μg g^–1^ dry matter, DM), organic Cl content (%), and Cl pool size (mg m^–2^), we used a mixed linear model for a split-plot experiment (JMP, SAS Institute, Carey, North Carolina). In the statistical model, habitat (dry, moist, or wet), transect (*Lillfjärden*, *Gällsboträsk*, *Labboträsk*, *Eckarfjärden*), and soil layer (litter, humus soil) or vegetation layer (ground, herb, wood, foliage) were treated as fixed factors, whereas the habitat by transect interaction (main plot) was treated as a random factor. Thus, for the test of the habitat effect, the 1 m^2^ field plot (and the associated tree) was used as the level of replication. For the tests of the effects of soil or vegetation layer, as well as the consistency of habitat effects among layers, layer within field plot (split-plot) were used as the level of replication. Cl measurements were log-transformed prior to the statistical analysis. Thus, the mean values (and standard errors) are given on a logarithmic scale. The habitat effect on biomass, litter, C/N ratio, pH, and soil water content were also examined by analysis of variance, using habitat and transect as fixed factors. We checked the model adequacy by ascertaining that the residuals were approximately normality distributed (Shapiro–Wilk *W* test > 0.1) and independent of the predicted values (by visual examination).

## Results and Discussion

### Soil Cl Concentrations Are Similar among Different Habitats, but the Fraction of Cl_org_ is Higher in Drier Habitats

**Figure 1:**
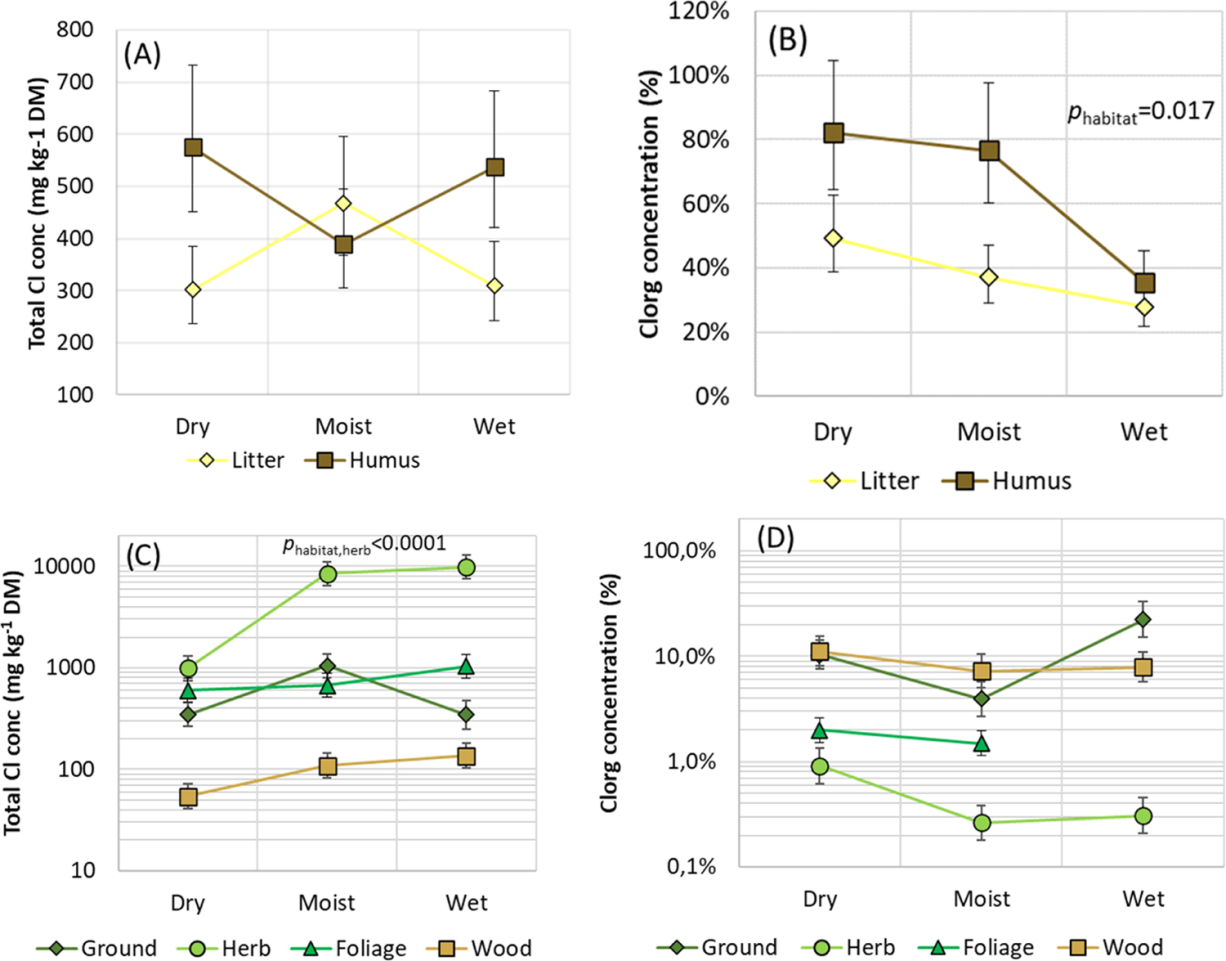
(A) Total Cl concentrations in soil, (B) percentage Cl_org_ in soil, (C) total Cl concentrations in biomass, and (D) percentage Cl_org_ in biomass along the hillslope gradient from dry upland coniferous forest (dry), moist coniferous forest (moist), to wet alder forest wetland (wet). Ground and herb refer to two separate vegetation layers, whereas foliage and wood are parts of the tree layer. Least-square means and standard errors on a logarithmic scale are shown.

The total Cl concentrations in litter and soil at the investigated sites spanned almost one order of magnitude, ranging from 220 μg g^–1^ DM (litter, wet alder site) to 1340 μg g^–1^ DM (in organic soil, wet alder site). Despite a large variation among sites, there were no clear tendencies for the total Cl concentration to change along the hillslope gradient and concentrations were similar in the two soil layers (Figure 1a). It is well-known that Cl is abundant in soils, primarily based on studies that have been conducted in well-drained soils (such as in the dry sites in this study). The total Cl concentrations of humus in the upland dry forest site and moist forest (Figure 1a) were in general higher, 390–580 μg g^–1^, than those reported from Southern Sweden, 99–458 μg g^–1^.^5,33^ Litter also had higher total Cl concentrations (300–470 μg g^–1^) than those reported from a study in France, 69–269 μg g^–1^.^16^ One possible reason for that could be that Forsmark is situated close to the Baltic Sea, thus having a maritime impact including an increased influence of atmospheric deposition of Cl from sea spray. Proximity to the coast and exposure to sea spray has previously been associated with an elevated soil Cl content.^11,33^ As Forsmark is located beneath the highest coastline, the soils are located on the former sea bed, and maritime deposits may still influence the soils.

In upland dry forest sites, the fraction of the total Cl that is Cl_org_ often exceeds 80% in humus, which is similar to that previously reported in upland forest soils.^1^ However, the fraction of soil Cl_org_ in humus varied along the hillslope (*p*_habitat_ = 0.017) and was substantially lower in wet habitats than at dry habitats (35 vs 82%, Figure 1b). There was a tendency that humus had a more pronounced decline of Cl_org_ downslope the gradient than litter had (Figure 1b), but the differences in response between humus and litter layers could have occurred due to chance (*p*_habitat×layer_ = 0.33). Thus, even if the total Cl concentrations are similar among habitats, there is a substantial variation in the chemical composition of soil Cl along the hillslope gradient and the fraction of Cl_org_ is higher in the drier habitats.

### High Cl_tot_ Concentrations in Biomass with Short Turnover Time

Cl was abundant in all types of vegetation investigated, but concentrations varied substantially among the different types of biomass samples (Figure 1c). In vascular plants, the highest total Cl concentrations were found in biomass having a fast turnover rate, such as foliage and annual herbs. For example, for trees, the total Cl concentration was considerably higher in foliage (746 μg g^–1^ DM) than in wood (92 μg g^–1^ DM). The total Cl concentrations were also high in the herb layer, and the concentrations increased along the hillslope gradient, from 990 μg g^–1^ DM in the dry habitat to 8430 and 9850 μg g^–1^ DM in the moist and wet habitats, respectively (*p*_habitat,herb_ < 0.001, Figure 1c). A similar, but less pronounced trend along the hillslope was also seen in wood (*p*_habitat,wood_ = 0.06, Figure 1c).

Chlorine is known to be ubiquitous in plant biomass but varies among species, as well as among different parts of the plants. Previously reported Cl concentration in foliage range from 183 to 820 μg g^–1^ DM,^16,34−37^ whereas that in wood varies between 6 and 95 μg g^–1^ DM.^35,37^ Thus, our results with eight times higher concentrations of Cl in foliage than in wood are consistent with previously reported results. Earlier studies indicate that the Cl concentration in deciduous foliage is lower than that in coniferous foliage. For example, Montelius et al.^16^ reported similar Cl concentrations in the foliage of different deciduous tree species and lower for Norway spruce growing on the same soil. In the current study, this pattern is not clear, as deciduous foliage (wet habitat, 1030 μg g^–1^ DM) tended to have a higher concentration than coniferous foliage (dry and moist habitat, 600 and 670 μg g^–1^ DM) (Figure 1c). At one of the wet sites, foliage from two deciduous species was collected, and the total Cl concentration in birch foliage was considerably lower, 380 μg g^–1^ DM (data not shown), than in alder foliage (1030 μg g^–1^ DM). This indicates that there can be large differences among foliage from different deciduous tree species at the same locations. No earlier studies on Cl concentrations in alder trees were found. For birch, foliage Cl^–^ levels of approximately 500 μg g^–1^ DM were observed by Edwards et al.^38^

### Species Composition Determines the Cl Concentration in the Herb Layer Rather Than Soil Cl Concentrations

The concentrations in the herb layer were even higher than those in tree foliage that previously has been shown to accumulate high Cl concentrations.^16,20,37^ Vegetation with annual above-ground plant parts, e.g., grass and herbs species, show high total Cl concentrations while plants with perennial plant parts, such as *Vaccinium vitis-idaea*, show lower concentrations (Figure S1). We also noted that the Cl concentrations of *Vaccinium myrtillus* and *Myrica gale* (which has a perennial stem) were higher than those in *Vaccinium vitis-idaea* having perennial parts only. This suggests an active regulation and limited Cl accumulation in perennial plant biomass, as compared to annual plants. It is also possible that surplus Cl is translocated to the annual biomass of the perennial plants. Cl^–^ is an essential micronutrient that has a direct role in, e.g., photosynthesis and stomatal regulation, and it is needed for ion balance and osmotic regulation.^39^ Thus, it is possible that the observed variation in biomass concentrations may be linked to conditions affecting one or several important plant functions.

The change in Cl concentration in herb vegetation along the hillslope was linked to a shift in species composition. That is, the low concentration in the dry habitat was associated with dwarf shrubs, which were rare in the moist habitats, and absent in the alder stands at the bottom of the hillslope. However, when dwarf shrubs were present in the moist habitats (at two sites), the concentration was similar to that in the dry upland habitat. Thus, it is likely that the differences in total Cl concentrations in vegetation is determined by the vegetation composition rather than the position along the hydrological gradient.

Cl^–^ was the dominating chlorine form in both tree (wood and foliage) and understory biomass. However, a substantial difference between vegetation layers was observed with respect to the fraction Cl_org_ (*p*_layer_ > 0.001, Figure 1d). That is, the Cl_org_ concentration ranged from approximately 10% in wood and mosses, over 2% in foliage, to less than 1% in the vegetation of the herb layer. There were no clear effects along the hillslope, but the pattern in the herb layer was inversely related to that of the total Cl concentration. That is, the percentage biomass Cl_org_ tended to be higher in the dry habitat (0.9%) as compared to the moist and wet habitats (0.26 and 0.31%).

The number of previous studies that have examined the form of Cl in plant tissue is limited but support a dominance of Cl^–^ over Cl_org_ in plant biomass.^16^ A relatively large fraction of Cl_org_ in wood, as compared to foliage, is in line with previously reported results for several different tree species, including oak, European beech, black pine, Douglas fir, and Norway spruce.^12^

### Cl Pools in Soil and Biomass

**Figure 2:**
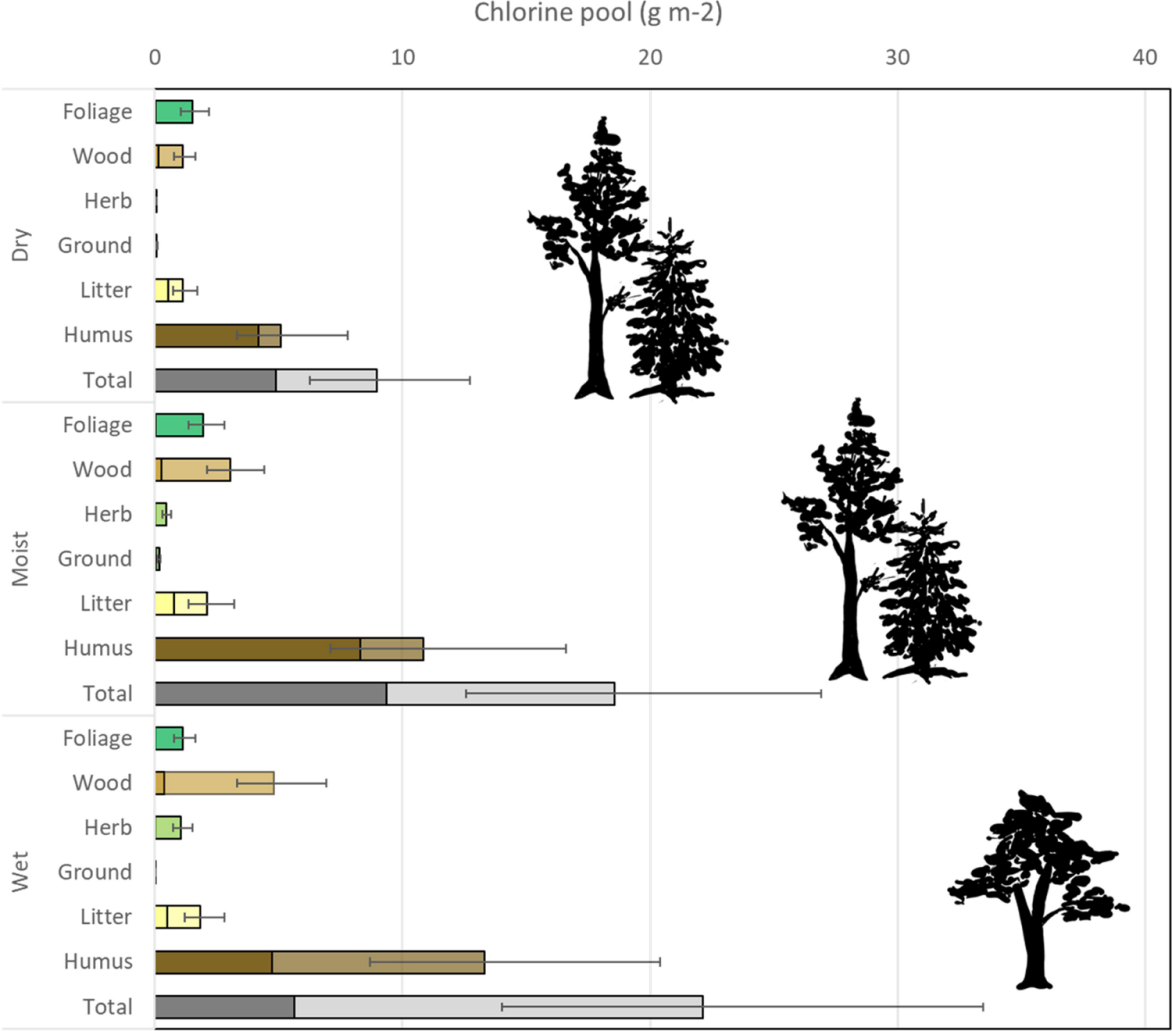
Summary of the total Cl pool among the different habitats. Dry: upland dry coniferous forest, moist: moist coniferous forest, and wet: wet alder forest wetland. Ground and herb refer to two separate vegetation layers, whereas foliage and wood are parts of the tree layer. The soil pool is represented by the litter and the humus layers (see text) and the total regards integrated ecosystem (see Table 3). Least-square means and standard errors are given. The Cl content is based on biomass assessments for respective classes and soil depth down to 0.4 m. The transparent part of the bars indicates Cl^–^ and the solid part is Cl_org_. The % Cl_org_ in foliage and ground vegetation was low, and this part of the Cl pool is not discernible in the figure. Least-square means and standard errors on a logarithmic scale are shown. Wet deposition in the area is 0.45 g Cl_tot_ m^–2^ y^–1^.

The total soil Cl pool down to the bottom of the humus layer ranged between 5 and 86 g m^–2^ (min-max) for individual sites, with typical values of 26 and 18 g m^–2^ (arithmetic and geometric means). The Cl content in humus soils was 5.5 times larger than that in litter (Figure 2, *p* < 0.001), and the content in both layers was twice as large in the wet and moist habitats as compared to the dry habitat (Figure 2). This shift could partly be attributed to a higher humus mass of soil layers downhill (Table 1). However, given the high variability in total Cl among sites, the difference between habitats could have occurred due to chance alone (*p* = 0.12 for the contrast between the dry and the other two habitats). The predominant form of soil chlorine in dry and moist habitats is Cl_org_ in humus (∼60%, Figures 1b; 2). The large fraction of Cl_org_ in soils at the dry habitats is in accordance with past findings for coniferous soils, 1.7–13 g Cl_org_ m^–2^ in areas dominated by Norway spruce in France.^4,16^ In the present study, the highest Cl_org_ content was observed at the dry sites and coincided with the highest content of soil carbon along the hillslope gradient (Table 2). This is consistent with previous observations from relatively dry upland areas, where the Cl_org_ content has been correlated to the carbon content in forest soils.^4^

The soil pool of Cl^–^ was clearly higher in the wet sites than in the dry and moist habitats (Figure 2). This pattern may be attributed to an increased influx of Cl^–^ due to old marine groundwater discharge at the wet sites. Field measurements from the site indicate that Cl^–^ concentrations are 80 times greater in shallow groundwater collected in permanent monitoring wells than in the precipitation (median value 60 mg L^–1^, *n* = 40,^40^). The concentration in deep groundwater collected in drilled boreholes in the bedrock is typically much higher in Forsmark.^40^ This means that the input from a discharge corresponding to less than 10 mm of shallow groundwater would equal the Cl^–^ load from the wet deposition.

The combined biomass contributed on average 30% of the total ecosystem pool of Cl, with stem biomass being the dominant component (geometric mean 2.5 g m^–2^, Figure 2). The dominant tree species in the dry and moist sites were pine and spruce, while alder dominated at all wet sites (Table S2). Tree foliage also contributed notably to the biomass pool (1.5 g m^–2^), whereas the Cl content in the herb and ground layers was low (0.3 and 0.05 g m^–2^, respectively). Total Cl pool in wood and in the herb layer clearly changed along the hillslope, with the Cl content being four and 20 times higher in moist and wet habitats than that in dry habitats (*p*-values for the linear contrasts comparing the mean of the moist and wet habitat with that of the dry habitat were 0.03 and <0.001 for wood and herbs, respectively, Student’s *t*-test). For wood, the shift reflects the combined response of a higher biomass and a higher Cl concentration in the wet habitat, whereas the primary driver for the response in the herb layer is the higher Cl concentration associated with herbs and grasses in the wet habitat. There have only been a few attempts to estimate the standing stock of Cl in biomass. Öberg et al.^41^ estimated the Cl pool in biomass, dominated by *P. sylvestris* to 2.1 g m^–2^ of which 0.1 g m^–2^ was Cl_org_. Montelius et al.^16^ estimated the total Cl in tree biomass for Norway spruce to approximately 2.5 g m^–2^. These estimates are similar to the Cl content in tree biomass found in the dry habitat in this study (2.6 g m^–2^).

### Turnover of Cl in Soil and Biomass

To get a better understanding of the Cl cycling in the examined habitats, the turnover times of the measured Cl pools were estimated (τ in Table 3 and Figure S2). Thus, the total Cl pools in deciduous biomass (the herb layer and alder foliage) were set to turnover in a year, the pools in evergreen foliage (coniferous needles and bryophytes) litter and humus were set to turnover within 10 years, whereas the total Cl pool in wood was expected to turnover in approximately a hundred years (Table 3). Assuming that total Cl pools were in a steady state, the yearly specific uptake of Cl^–^ (including chlorination) was then calculated for each pool by dividing pool sizes with turnover times (k in Table 3, see Materials and Methods).

**Table 3:** Turnover Time (τ), Transfer Rates (*k*), and Cl Fluxes of Ecosystem Pools in Three Habitats along a Hillslope Gradient^a^

		*k*_Cl^–^_ (year^–1^)			Cl flux (g m^–2^ year^–1^)		
pool	(year)	dry	moist	wet	dry	moist	wet
foliage	7.5, 1^b^	0.14	0.07	0.11	0.20	0.26	1.13
wood	150, 100^b^	0.005	0.005	0.005	0.01	0.02	0.05
herb layer	1	0.03	0.12	0.11	0.05	0.46	1.05
ground layer	3	0.02	0.01	0.0003	0.02	0.06	0.003
litter (Cl_org_)	8, 4^b^	0.05	0.03	0.01	0.07	0.10	0.13
humus (Cl_org_)	5	0.56	0.43	0.10	0.8	1.7	0.9
export (Cl^–^)	I/[Cl^–^]	0.30	0.12	0.09	0.45	0.45	0.90

a Turnover time is based on the literature with respect to organic material in the respective pools. The humus pool is an exception as it explicitly represents the organic Cl pool in this soil layer. The rate constant (*k*) represents the fraction of the soil Cl pool that is immobilized in internal pools or exported from the system each year. Calculations assuming steady-state conditions, implying that Cl fluxes into and out of the pools are in balance. For the dry and moist habitats, τ_export_ was calculated assuming that the Cl import was dominated by the wet deposition (*I* = 0.45 g m^–2^ year^–1^). Wet habitats were assumed to receive an equal amount of chloride through groundwater discharge in addition to wet deposition (I = 0.9 g m^–2^ year^–1^, see text for discussion).

b Denotes wet habitats.

Considering all pathways of uptake and export (i.e., the sum of all rate constants *k* in Table 3), the residence time of Cl^–^ ranged from 11 months in the dry and moist habitats to 2.4 years in the wet habitat. Moreover, a comparison of rate constants suggests that internal uptake was two to five times as likely as export from the system. Although most of the internally cycled Cl will be returned to the soil Cl^–^ pools within a few years, the uptake is likely to dampen short-time fluctuations of the soil Cl^–^ pool. The estimated largest internal fluxes of Cl^–^ (>1 g m^–2^ year^–1^) were attributed to uptake by herbaceous vegetation and alder foliage in the wet habitat as well as to chlorination of humus in all habitats.

When the system is in a steady state, the ratio between the soil Cl_org_ and Cl^–^ pools are directly reflecting the ratio of chlorination to dechlorination rates. Thus, it appears that the ratio of the two processes is clearly shifted along the hillslope gradient. If the turnover time of Cl_org_ in humus is similar in all three habitats (as postulated), then the specific chlorination rate decreases by a factor of five along the hillslope gradient; from approximately 0.5 year^–1^ in the dry and moist habitats to 0.1 year^–1^ in the wet habitat (Table 3). However, it is also possible that the relatively low content of Cl_org_ in the wet habitats reflects an increased rate of dechlorination, as it has been suggested that the rate of this process is enhanced under anaerobic conditions.^8,26^

The dominating formation processes of organochlorines are biotic;^9^ however, the specific formation processes are still not understood. A common hypothesis is that Cl_org_ is formed during the degradation of organic matter through the (enzymatic) formation of reactive chlorine. The two main substrates for this process are organic matter and Cl^–^. In the pH interval 3–5, the formation of Cl_org_ decreases with increasing pH.^25,42^ There was a considerable shift in pH along the hillslope where soil pH increased successively from the upland coniferous forest to the wet alder forest sites, with averages of 3.2 (pH_KCl_) and 6.0 for the dry and wet sites, respectively (Table 1). Nitrogen has been shown to have a hampering effect on chlorination,^26,27^ but the nitrogen content in the different habitats showed small variability (Table 1) and thus did not appear to be a major driver.

Temporal immobilization of Cl in the litter pool by chlorination only accounted for between 4% and 6% of the total internal uptake of Cl^–^. In the dry and moist sites, the areal chlorination rates calculated from mass balance (0.07–0.10 g m^–2^ year^–1^, Table 2) were in the same order as previously measured in spruce litter (0.035–0.05 m^–2^ year^–1^).^43,44^ The calculated uptake of Cl in stem wood was even slower, and for all three habitats, it was approximately an order of magnitude lower than the accumulation in litter (Table 3). However, the cumulative uptake over the last century has resulted in a substantial pool of Cl in wood (∼20 to 30% of the total ecosystem storage). The turnover of this pool is slow (i.e., 100–150 years).

On a larger landscape scale, deposition of Cl^–^ is regarded to be the major factor explaining soil Cl_org_ concentrations and soil Cl pools.^33^ In this study, we have shown that the position of soils along a hillslope may be an additional factor that influences the Cl pools and suggest that long-term patterns in chlorination and dechlorination may be a key factor behind the observed patterns. We have also shown that biological processes are a key driver behind the main fluxes of Cl in the examined habitats and suggest that production and species composition of both the tree and the herb layer may have a major influence on these patterns. In specific, we point out that wet habitats at the base of hillslopes are areas that differ in several aspects of Cl cycling from drier upland sites. Mass-balance modeling suggests that wet habitats are hot spots for Cl cycling, and long-term immobilization by net chlorination appears to be hampered at these sites. These habitats are also most likely to be influenced by loads of Cl^–^ from the discharge of groundwater. Our study demonstrates that it is necessary to characterize Cl^–^ and Cl_org_ uptake and storage in the common habitats and associated vegetation communities to estimate overall landscape Cl cycling and residence times. The variation between sites within habitats was substantial in this study and further work is needed to consolidate our findings. Such studies should preferably link direct measurements of chlorination and dechlorination rates to environmental factors in a landscape context and aim for direct quantification of soil Cl^–^ turnover in different habitats.

## Supporting Information Available

Additional information on geographical position and elevation of sampling sites; vegetation characteristics of the sites; Cl concentrations in understory vegetation; conceptual figure of Cl transfer rates in soil and biomass; and transfer rate estimates (PDF)

## Dedication

Dedicated to the memory of Anders Löfgren.
