# Supplementary material for: Chlorine Distribution in Soil and Vegetation in Boreal Habitats along a Moisture Gradient from Upland Forest to Lake Margin Wetlands
Source: Environ Sci Technol. 2023 Jul 20;57(30):11067–74. doi: 10.1021/acs.est.2c09571 (PMC10399286; doi:10.1021/acs.est.2c09571)
Supplement: Supplementary file 1 — es2c09571_si_001.pdf [file es2c09571_si_001.pdf]

Supporting information

Chlorine distribution in soil and vegetation in boreal habitats along a moisture gradient from upland forest to lake margin wetlands

Svensson Teresia[1]*, Löfgren Anders[2], Saetre Peter[3], Kautsky Ulrik[3], Bastviken David[1]

1. Department of Thematic Studies - Environmental Change, Linköping University

2. EcoAnalytica, Slalomvägen 28, 129 49 Hägersten, Sweden

3. Swedish Nuclear Fuel and Waste Management Co. (SKB), P.O. Box 3091, 169 03 Solna, Sweden

*Corresponding author. teresia.svensson@liu.se

Content

Supplementary text: Transfer rate estimates 2

Supplementary table: Table S1. Geographical position and elevation of sampling sites....... 3

Supplementary table: Table S2. Vegetation characteristic........ 4

Supplementary figure: Figure S1. Cl concentrations in understory vegetation 5

Supplementary figure: Figure S2. Conceptual figure of Cl transfer rates in soil and biomass 6

References 7

Supplementary text: Transfer rate estimates

For each habitat, the transfer rates from the soil Cl^- pool to each biomass pool were calculated assuming (i) that plants only take up Cl^- (i.e. negligible amounts of Cl_{org}), (ii) that the turnover of Cl follows that of biomass so that the ratio of total Cl in biomass ($Cl_{tot,i}$) to available Cl^- in the soil (Cl^-_{soil}) represents the net plant uptake over the whole biomass turnover time and therefore needs to be divided with the turnover time in years to generate the fraction of Cl^-_{soil} that is transferred to biomass each year, and (iii) that the Cl pools were in steady-state. Thus, the rate constant k (fraction of soil Cl^- transferred year^{-1}) for each biomass pool i becomes:

$$k_i = \frac{Cl_{tot,i}}{\tau_i \cdot Cl^-_{soil}}$$

where $Cl_{tot,i}$ is the total biomass Cl content (g m^{-2}), τ_i is the biomass turnover time (year), Cl^-_{soil} is the soil Cl^- content in humus and litter (g m^{-2}) and i is an index for biomass pools (i.e. foliage, stem wood, herb layer and ground layer). Herb layer vegetation and deciduous litter was assumed to turn over annually. Ground vegetation (e.g. mosses) was assumed to turnover in 3 years [1] and references within, whereas coniferous foliage was assumed to turnover in 7.5 years (average of pine and spruce needles, [2, 3]. The turnover of wood included both tree mortality and wood decomposition and was set to 150 and 100 years for coniferous trees and alder respectively [4, 5]. Litter turnover was calculated for each habitat by dividing the litter mass with the foliage production (Table 3, where production equals foliage biomass divided by its turnover time). Thus, the litter turnover times were set to 8 years for the dry and moist habitat, and 4 years for the wet habitat.

The turnover time for Cl_{org} in humus was calculated from measured pools of Cl_{org} in humus and the soil Cl^- pool at the dry sites and previously measured rates of chlorination in upland forest sites [4, 5, 6, 7]. Assuming that chlorination and dechlorination was in equilibrium, the turnover of Cl_{org} in humus was calculated to be approximately 5 years (corresponding to a specific chlorination rate of ~ 0.001 d^{-1}). This turnover time suggests that dechlorination is associated with the decomposition of both labile and more recalcitrant residues of soil organic matter (SOM). For simplicity the turnover time was applied to all three habitats, and rate constants for chlorination were calculated for each habitat as described above. Finally, the turnover time and rate constant of the soil Cl^- pool associated with export from the system was calculated from the soil Cl^- pool size and the Cl^- load (g m^{-2} years^{-1}).

Supplementary table: Table S1. Geographical position and elevation of sampling sites

Geographical position and elevation of sampling sites. The distance to the groundwater level and the time since the site emerged from the sea were estimated from the present height above the water surface of the closest lake and the height above the sea level, respectively. Dry: upland dry coniferous forest, Moist: moist coniferous forest, and Wet: wet alder forest wetland (Sweref 99 18 00). The elevations of the habitat sampling site, and the elevation above downstream lake level, were estimated using a laser-based digital elevation map of the area [8]. The time since the habitat sampling site emerged from the sea due to land uplift was estimated based on the current elevation above sea level (RH2000) and a model describing the historic sea-level change in Forsmark [9].

Locations	Sampling Site	North-South SWEREF 99 18 00	East-West SWEREF 99 18 00	Elevation RH2000 (m)	Depth to water (m)	Time since isolation (y)
**Lillfjärden**	Dry	6696915.4	162354.7	7.8	9.8	1290
	Moist	6696770.1	162484.5	1.8	1.5	350
	Wet	6696712.8	162544.1	-0.2	0.2	10
**Gällsboträsk**	Dry	6697136.4	159339.2	5.9	5.6	1000
	Moist	6697095.9	159372.1	2.9	3.8	540
	Wet	6696989.1	159490.1	1.7	0.0	340
**Labboträsk**	Dry	6697775.2	158879.6	7.0	5.2	1180
	Moist	6697748.2	158944.9	4.0	3.8	700
	Wet	6697592.9	158921.9	3.7	1.8	660
**Eckarfjärden**	Dry	6695773.5	160584.6	7.8	1.9	1300
	Moist	6695632.9	160648.4	6.2	1.0	1050
	Wet	6695645.4	160695.6	5.2	0.1	900

Supplementary table: Table S2. Vegetation characteristic

Vegetation characteristics of the three different sites. Dry: upland dry coniferous forest, Moist: moist coniferous forest, and Wet: wet alder forest wetland. dbh= diameter at breast height

Properties	Transect	Dry	Moist	Wet
**Vegetation type[a]**	All	Spruce forest of bilberry type	Spruce forest of low herb type	Wet alder forest of herb type
**Dominant tree closest to sampling plot**	*Lillfjärden*	*Picea abies*	*Picea abies/Pinus sylvestris*	*Alnus glutinosa*
	Gällsboträsk	*Picea abies*<br>*Pinus Sylvestris*	*Picea abies*<br>*Pinus Sylvestris*	*Alnus glutinosa*<br>…
	Labboträsk	*Pinus Sylvestris*<br>*Picea abies*	*Picea abies*<br>*Pinus Sylvestris*	*Alnus glutinosa Pinus Sylvestris*
	Eckarfjärden	*Picea abies*<br>*Pinus Sylvestris*	*Picea abies*<br>*Pinus Sylvestris*	*Alnus glutinosa*<br>*Betula pubescens*
**Tree density (no trees/100 m^{-2})**	*Lillfjärden*	11	7	26
	Gällsboträsk	10	27	24
	Labboträsk	12	19	15
	Eckarfjärden	10	10	58
**Average tree height (m)[b]**	*Lillfjärden*	16	16	14
	Gällsboträsk	23	21	15
	Labboträsk	17	17	13
	Eckarfjärden	18	21	10
**Average dbh (m)[b]**	*Lillfjärden*	0.19	0.20	0.18
	Gällsboträsk	0.34	0.32	0.22
	Labboträsk	0.25	0.20	0.25
	Eckarfjärden	0.26	0.30	0.14
**Basal area (m^2/ha)**	*Lillfjärden*	3100	2100	2300
	Gällsboträsk	3100	3400	3400
	Labboträsk	2500	4100	3100
	Eckarfjärden	3200	4200	4500
**Tree age dbh (1 tree)**	*Lillfjärden*	59[a]	88[a]	45
	Gällsboträsk	88	85	62
	Labboträsk	155[a]	101	139[i]
	Eckarfjärden	132	118	62
**Typical herb layer species[c]**	*Gällsboträsk*	*Vaccinum myrtillus, Vaccinum vitis-idaea*	*Melica nutans, Dactylis glomerata*	*Carex appropinquata, Filipendula ulmaria*
	Lillfjärden	*Vaccinum vitis-idaea, Vaccinum myrtillus*	*Hepatica nobilis, Convallaria majalis*	*Eupatorium canabinum, Filipendula ulmaria*
	Labboträsk	*Vaccinum myrtillus, Vaccinum vitis-idaea*	*Vaccinum myrtillus, Calamagrostis canescens*	*Phragmites australis, Carex diandra*
	Eckarfjärden	*Vaccinum vitis-idaea, Vaccinum myrtillus*	*Lysimachia vulgaris, Vaccinum vitis-idaea*	*Filipendula ulmaria, Thelypteris palustris*
**Dominating ground vegetation layer species**	*Lillfjärden*	*Hylocomium splendens, Pleurozium schreberi*	*Hylocomium splendens, Pleurozium schreberi,*	–
	Gällsboträsk	*Hylocomium splendens, Ptilium crista-castrensis*	*Dicranum majus, Hylocomium splendens*	*Herzogiella seligeri, Mnium sp.*
	Labboträsk	*Hylocomium splendens, Pleurozium schreberi*	*Herzogiella crista-castrensis, Hylocomium splendens*	*Calliergonella. cuspidate,*
	Eckarfjärden	*Hylocomium splendens, Rhytidiadelphus triquetrus*	*Rhytidiadelphus triquetrus, Pleurozium schreberi*	*Herzogiella seligeri, Mnium sp.*

a. *(Påhlsson 1998)*
b. *N=4-6*
c. *See specific description if the species abundance in Appendix 5.*

i= tallest tree non-dominat species (Pinus sylvestris)

Supplementary figure: Figure S1. Cl concentrations in understory vegetation

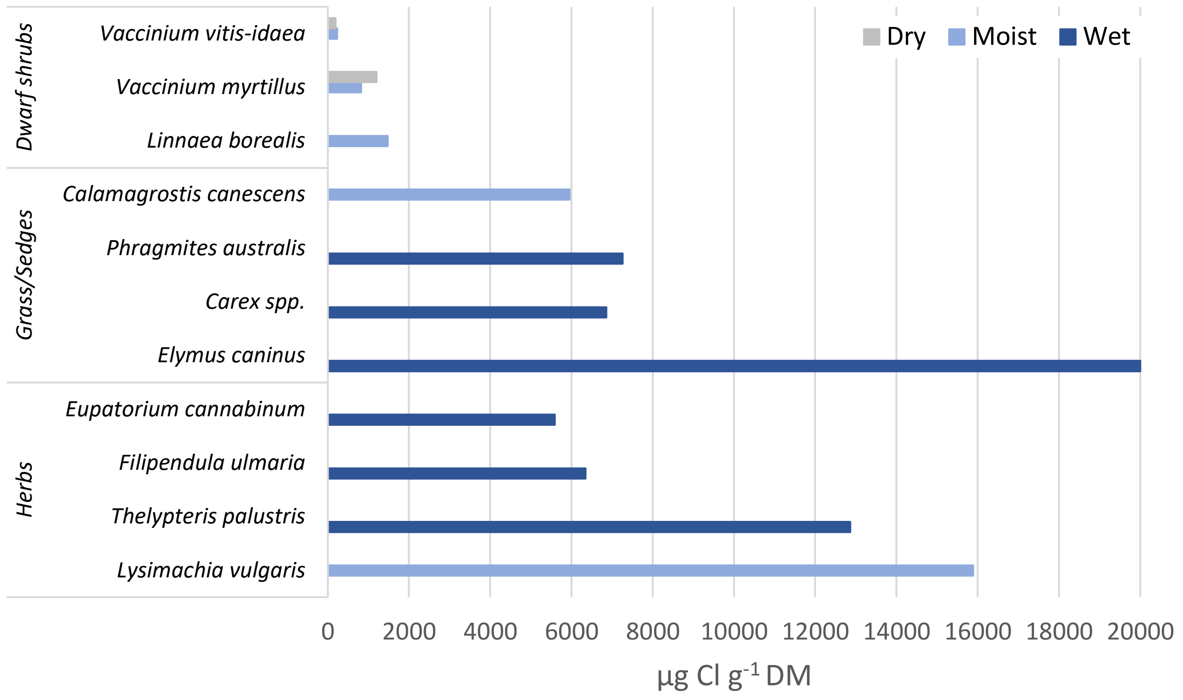

Average total Cl concentrations in sampled understory vegetation in the three different habitats (see methods for definition of dry, moist and wet habitats, respectively). For species where more than one sample was collected, the geometric mean is given.

Supplementary figure: Figure S2. Conceptual figure of Cl transfer rates in soil and biomass

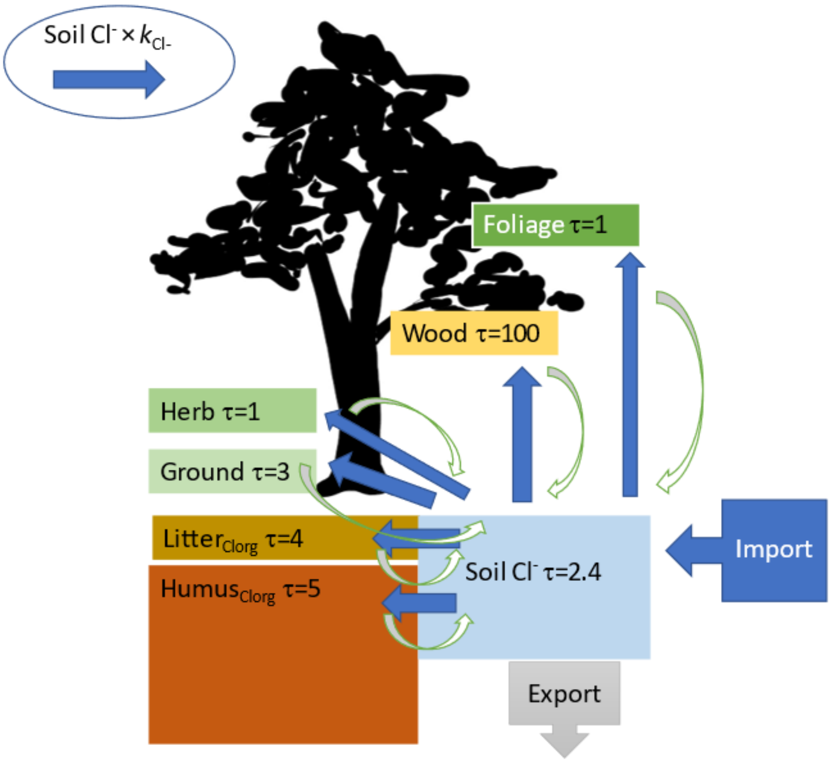

References

1. Liski, J., Lehtonen, A., Palosuo, T., Peltoniemi, M., Eggers, T., Muukkonen, P., & Mäkipää, R. (2006). Carbon accumulation in Finland's forests 1922-2004 - An estimate obtained by combination of forest inventory data with modelling of biomass, litter and soil. Annals of Forest Science, 63(7), 687-697. https://doi.org/10.1051/forest:2006049
2. Muukkonen, P., & Lehtonen, A. (2004). Needle and branch biomass turnover rates of Norway spruce (Picea abies). Canadian Journal of Forest Research, 34(12), 2517-2527. https://doi.org/10.1139/x04-133
3. Muukkonen, P., *Needle biomass turnover rates of Scots pine (Pinus sylvestris L.) derived from the needle-shed dynamics.* Trees, 2005. **19**(3): p. 273-279.
4. Busing, R. T. (2005). Tree mortality, canopy turnover, and woody detritus in old cove forests of the southern Appalachians. Ecology, 86(1), 73-84. https://doi.org/10.1890/04-0410
5. Stephenson, N. L., & Van Mantgem, P. J. (2005). Forest turnover rates follow global and regional patterns of productivity. Ecology Letters, 8(5), 524-531. https://doi.org/10.1111/j.1461-0248.2005.00746.x
6. Bastviken, D., Svensson, T., Karlsson, S., Sandén, P., & Öberg, G. (2009). Temperature sensitivity indicates that chlorination of organic matter in forest soil is primarily biotic Environmental Science & Technology, 43(10), 3569-3573.
7. Bastviken, D., Thomsen, F., Svensson, T., Karlsson, S., Sandén, P., Shaw, G., Matucha, M., & Öberg, G. (2007). Chloride retention in forest soil by microbial uptake and by natural chlorination of organic matter. Geochimica et Cosmochimica Acta, 71(13), 3182-3192.
8. Montelius, M., Svensson, T., Lourino-Cabana, B., Thiry, Y., & Bastviken, D. (2016). Chlorination and dechlorination rates in a forest soil - A combined modelling and experimental approach [Article]. Science of the Total Environment, 554-555, 203-210. https://doi.org/10.1016/j.scitotenv.2016.02.208 Muukkonen, P. (2005). Needle biomass turnover rates of Scots pine (Pinus sylvestris L.) derived from the needle-shed dynamics. Trees, 19(3), 273-279. https://doi.org/10.1007/s00468-004-0381-4
9. Öberg, G., Börjesson, I., & Samuelsson, B. (1996). Net change in organically bound halogens in relation to soil pH. Water, Air and Soil Pollution, 89, 351-361.
10. Petrone J, Strömgren M, 2020. Baseline Forsmark – Digital elevation model. SKB R-17-06, Svensk Kärnbränslehantering AB. Stockholm
11. Påsse T, 2001. An empirical model of glacio-isostatic movements and shore-level displacement in Fennoscandia. SKB R-01-41, Svensk Kärnbränslehantering AB. Stockholm
